# Supplementary material for: Structural colour enhanced microfluidics
Source: Nat Commun. 2022 May 19;13:2281. doi: 10.1038/s41467-022-29956-4 (PMC9120135; doi:10.1038/s41467-022-29956-4)
Supplement: Supplementary file 1 — Supplementary Information [file 41467_2022_29956_MOESM1_ESM.pdf]

Supplementary Information for

Structural Colour Enhanced Microfluidics

Detao Qin[1,2], Andrew H. Gibbons[1], Masateru M. Ito[1,2]*, Sangamithirai Subramanian Parimalam[1], Handong Jiang[1,2], H. Enis Karahan[1], Behnam Ghalei[1,2], Daisuke Yamaguchi[1,2], Ganesh N. Pandian[1], Easan Sivaniah[1,2]*.

Affiliations:
[1]Institute for Integrated Cell-Material Sciences (iCeMS), Kyoto University of Advanced Study, Kyoto University, 606-8501, Kyoto, Japan.
[2]Department of Molecular Engineering, Kyoto University, 606-8501 Kyoto, Japan.
*Correspondence to: mito@icems.kyoto-u.ac.jp (M.I.); esivaniah@icems.kyoto-u.ac.jp (E.S.)

This file includes

- Supplementary Figures 1–13.
- Supplementary Tables 1–6.
- Supplementary Discussion.
- Supplementary References

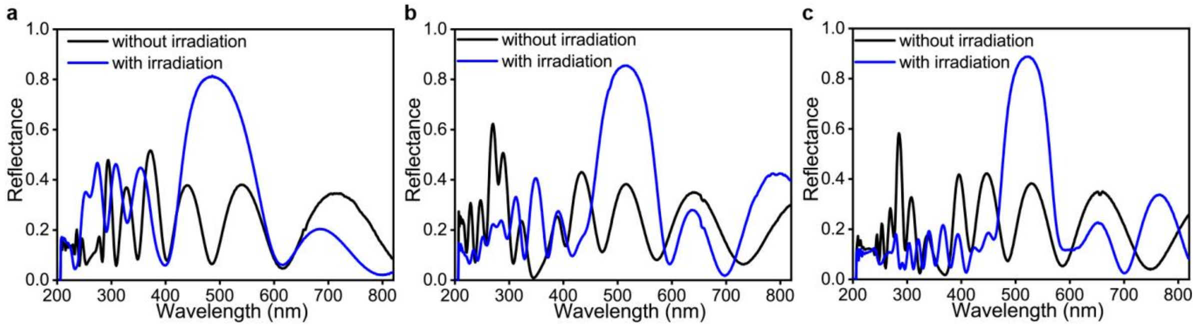

Supplementary Fig. 1 | Example reflectance spectra for OM microfluidic films. Polymer is **a**, polystyrene (PS), **b**, poly(methyl methacrylate) (PMMA), and **c**, polycarbonate (PC). These OM microfluidic films were printed on silicon wafer using stainless steel stencil as the shadow mask. Photoinitiator is 9,10-phenanthrenequinone (PQ) for (**a**) and 4,4′-bis-(diethylamino)-benzophenone (BDABP) for (**b**, **c**). Photocrosslinking was conducted in a custom oven (λ_i, 375 nm). After the development step, a periodic structure is formed at the irradiated region of the films, which is indicated by the Bragg peak on their reflectance spectra (blue curves); in contrast, the non-irradiated region of these films shows a thin film reflectance profile (black curves). Bragg peak location can be tuned by illumination wavelength (λ_i) and other experimental conditions.

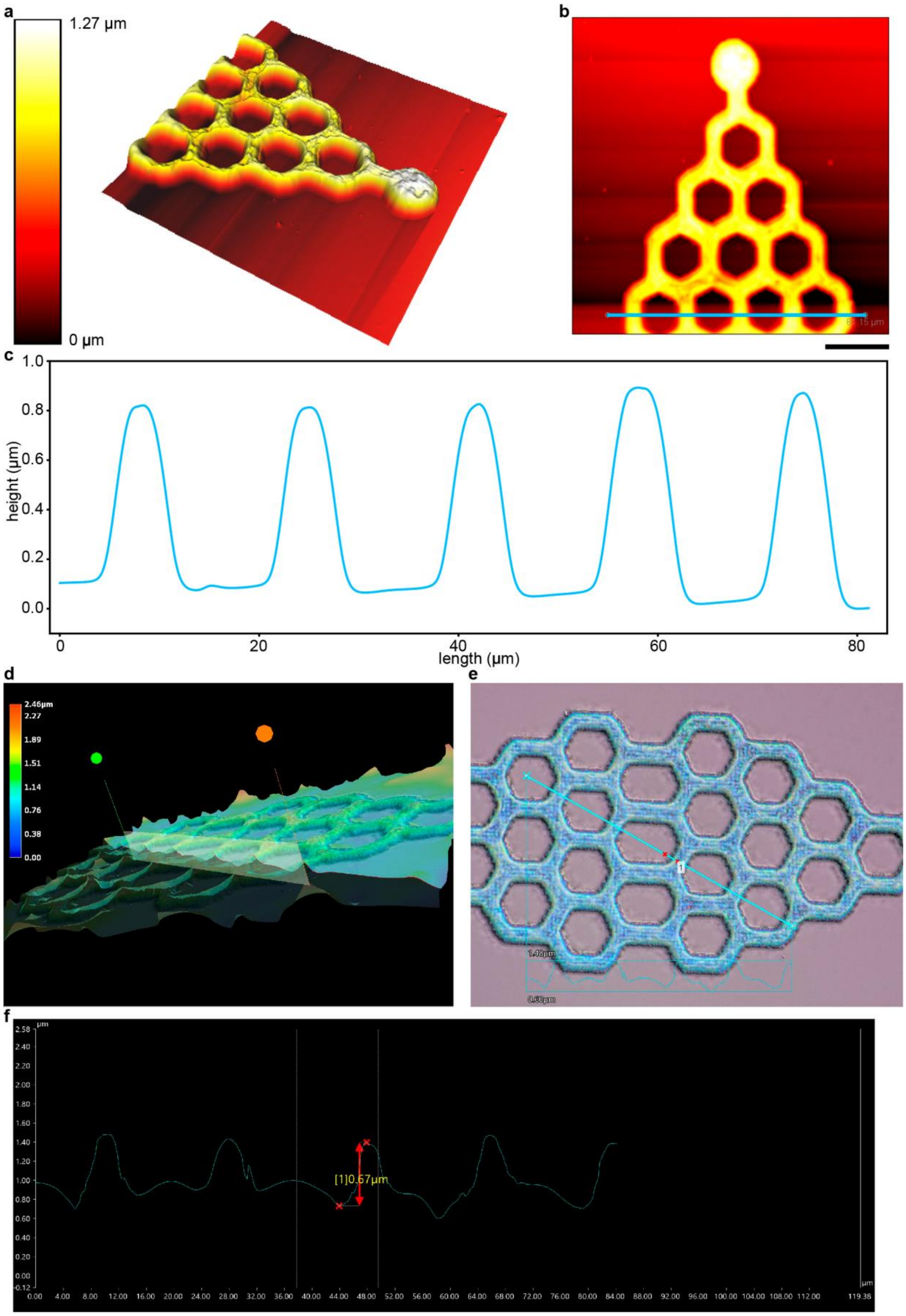

Supplementary Fig. 2 | OM channel topography. a–c, OM channel topography characterized by AFM, wherein (**a**) 3D image, (**b**) 2D image, and (**c**) the corresponding height profile. **d–f**, OM channel topography characterized by a Keyence digital microscope, wherein (**d**) 3D image, (**e**) 2D image, and (**f**) the corresponding height profile. Both methods confirm height expansion of the channel compared to the non-irradiated region of the same film. The sample was made using PS/PQ on silicon wafer using a micro-LED instrument.

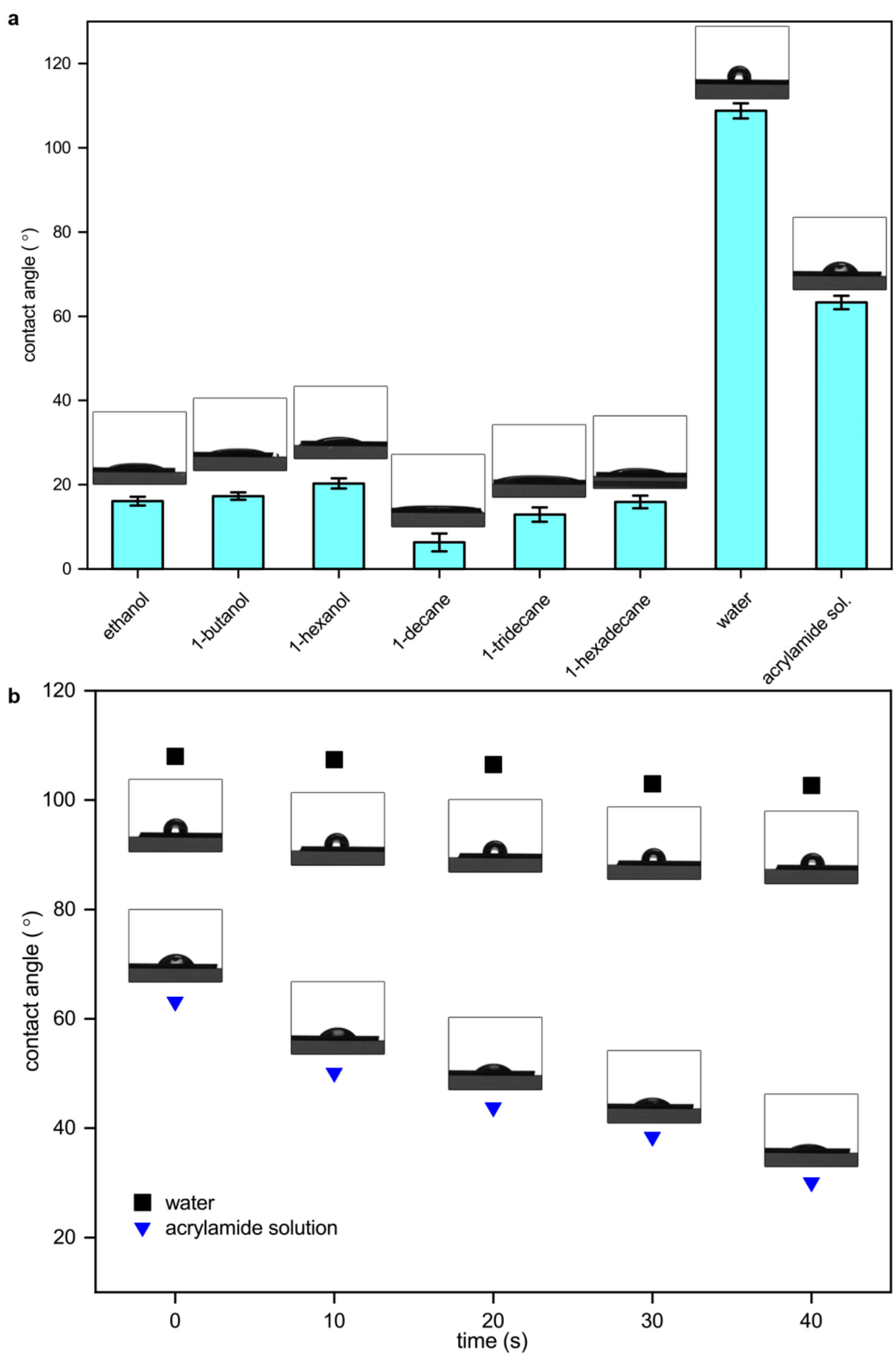

Supplementary Fig. 3 | Contact angles of OM films. a, The contact angle of OM film surface for various liquids. The contact angles were recorded by a sessile droplet method at the initial moment when the droplet fully settled on the film (time = 0 s). Reported values are the arithmetic mean and standard deviation (S.D.) from three samples. **b**, The change of contact angle with time for pure water and acrylamide aqueous solution (concentration: 10 wt%), respectively. The OM films were prepared using polystyrene (PS)/9,10-phenanthrenequinone (PQ) on silicon wafer. The formed OM structure has a Bragg peak of 670 ±20 nm (LED λ_i = 405 nm). The volume of probe liquids is 1.5 µl.

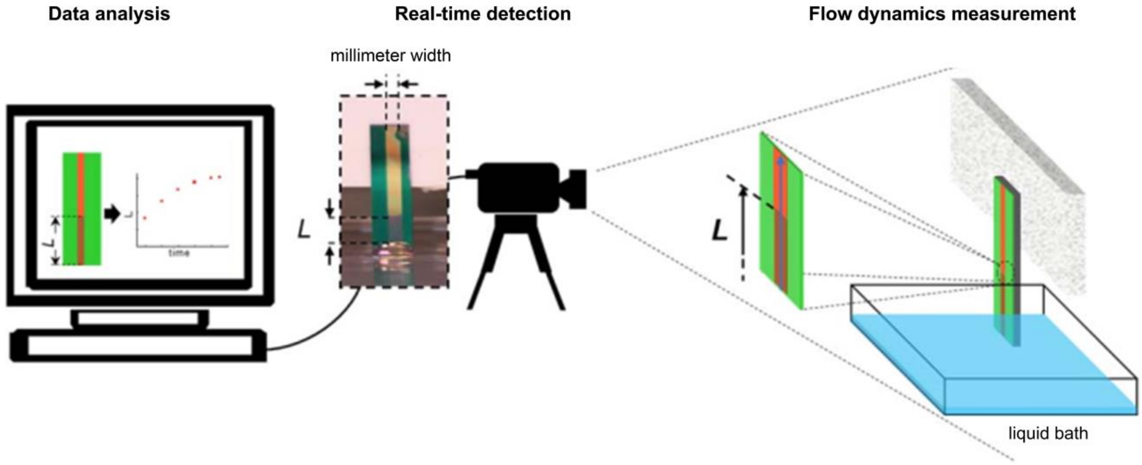

Supplementary Fig. 4 | The experimental setup using digital single-lens reflex (DSLR) camera for measuring flow dynamics in OM macrochannels. The details about this experimental setup have been provided in the Methods section.

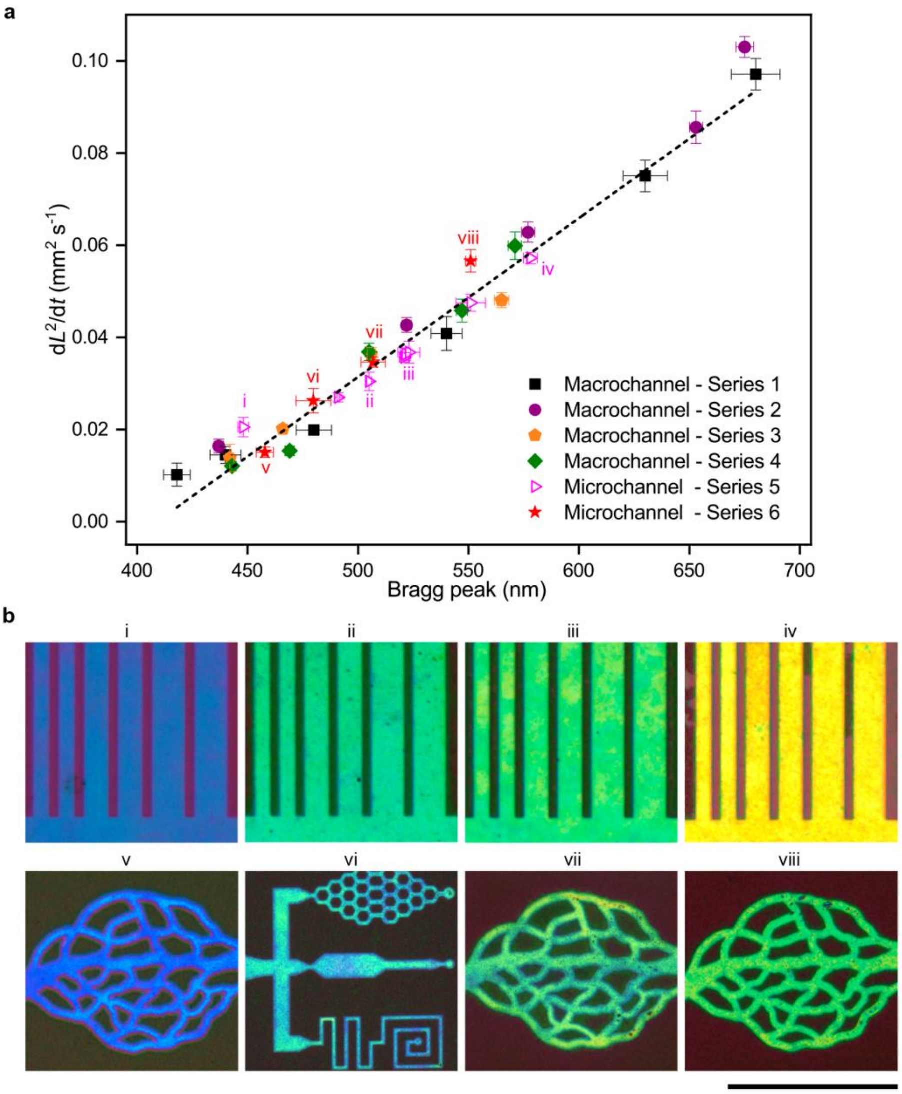

Supplementary Fig. 5 | Structural colour and fluidic property are coupled in OM channels. a, The measurement of dL^2/dt for OM channels printed at different length scales. This figure is the same as Fig. 3d in the main text. Series 1, 2 are flow measurement from 3-mm-width channels; series 3, 4 are flow measurement from 5-mm-width channels. Series 5, 6 are flow measurement from micron-sized channels (features from 5 to 40 microns). The channels, prepared under different photoinitiator and illumination conditions, are specified in Supplementary Table 3. Bragg peak of microchannels was estimated through converting microscope photo hue value to wavelength value (see the calibration in Supplementary Fig. 6). Here, macrochannels were fabricated in straight-line shape using stainless steel stencil as the shadow mask (Series 1–4). Microchannels were fabricated using a micro-LED illumination instrument (Series 5&6). The liquid is *n*-hexadecane and the substrate is silicon wafer. Error bars are S.D. across 3 measurements. **b**, Optical microscope photos for the fabricated microchannels (Series 5&6). Scale bar, 200 μm.

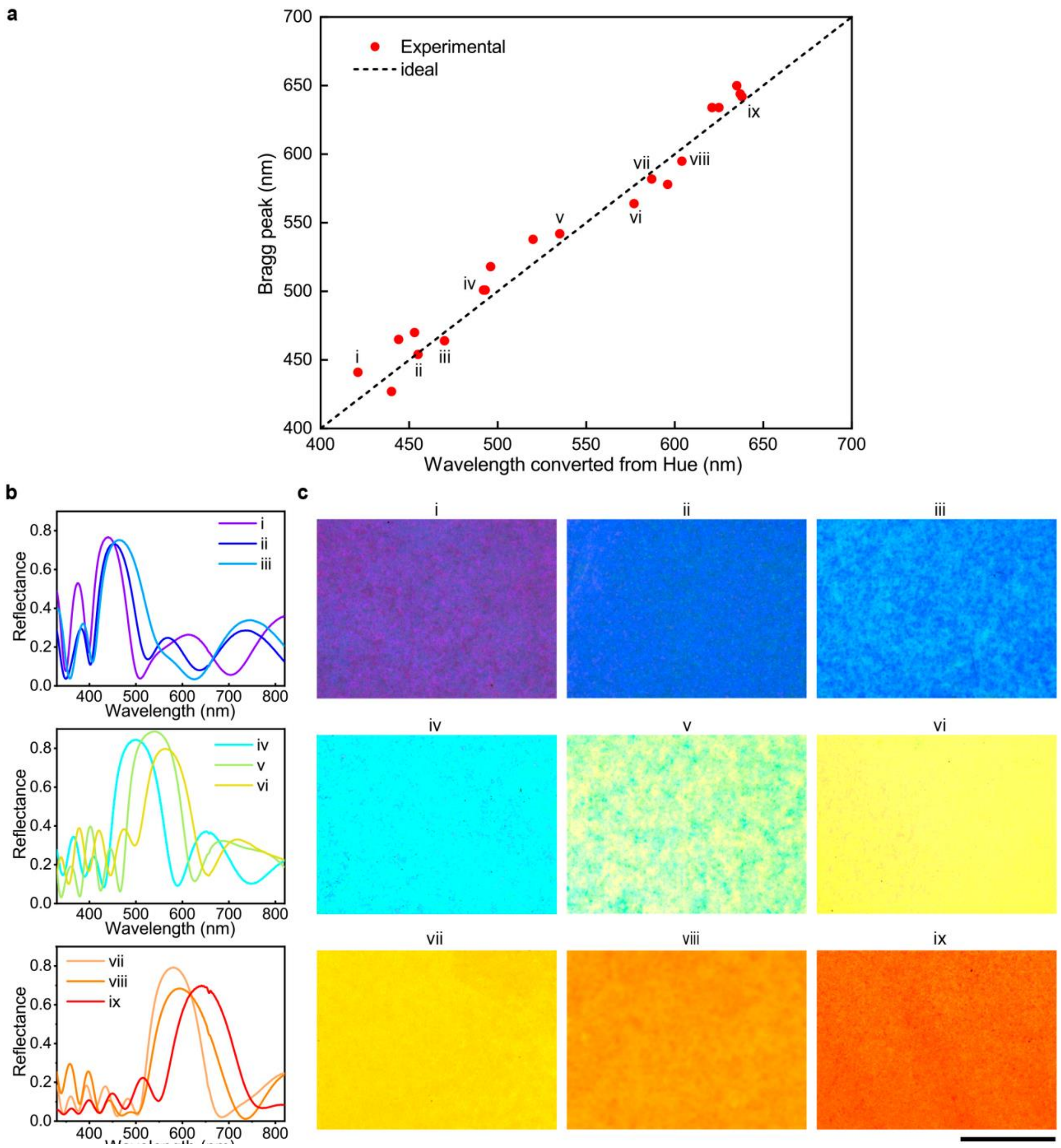

Supplementary Fig. 6 | Calibration for converting microscope photo hue value to Bragg peak. a, The calibration plot for converting microscope photo hue value to Bragg peak. **b**, The example reflectance spectra for OM channels. **c**, The corresponding microscope photos for OM channels. Scale bar, 500 μm. The Bragg peak of these channels was measured by a spectrometer (MCPD-3700, Otsuka Electronics). Correspondingly, photos were recorded by an optical microscope (Axioscope A1 MAT, Carl Zeiss, white balanced prior to photo-taking). The wavelength value is converted from microscope photo hue value according to the visible light spectrum chart, using *ImageJ* software (version 1.52p). The dashed line in the calibration plot displays the ideal case where wavelength value equals to the Bragg peak. The red dots denote the experimental results, which shows the error between spectrometer measured Bragg peak and microscope recorded wavelength value is 2.2% on average and less than 4.5% for all the OM channels fabricated. These results justify using the wavelength value of the microscope photo to estimate Bragg peak. This produces a convenient way for studying OM microchannels, especially when the channel print area is tiny and thus the direct measurement of Bragg peak using the spectrometer becomes difficult.

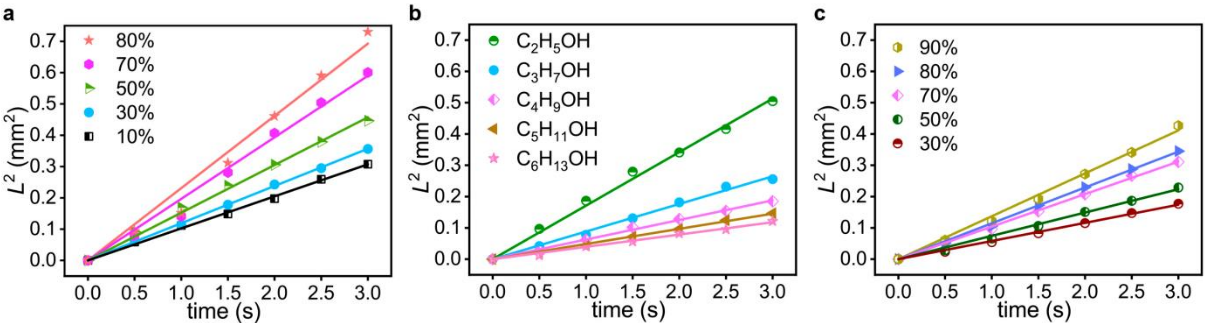

Supplementary Fig. 7 | L^2 versus t for (a) alkane mixtures, (b) pure alcohols, and (c) alcohol mixtures. The percentage numbers in (a) refer to the mole fraction of *n*-decane in the binary mixture of *n*-decane and *n*-hexadecane. The percentage numbers in (c) refer to the mole fraction of ethanol in the binary mixture of ethanol and *n*-hexanol. The OM samples were made using polystyrene (PS)/9,10-phenanthrenequinone (PQ) on silicon wafer. The illumination LED light (λ_i) is 405 nm and the Bragg peak of the formed OM structure is 670 ± 20 nm. Flow velocities were measured by an optical microscope (Axioscope A1 MAT, Carl Zeiss).

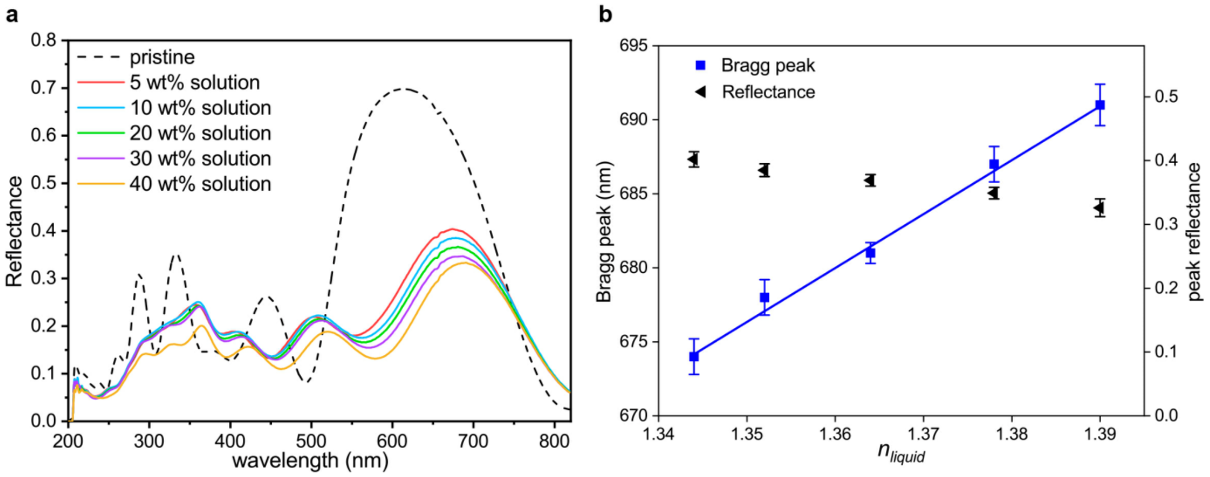

Supplementary Fig. 8 | Refractive index based sensing of aqueous solutions with different acrylamide concentrations. a, The reflectance spectrum of OM microfluidics for the aqueous solutions of different acrylamide concentrations. **b**, The corresponding Bragg peak location and reflectance versus the refractive index of acrylamide aqueous solutions. Refractive index (n_{liquid}) of acrylamide aqueous solutions with different concentrations is obtained from literature[1]. The OM samples were made using polystyrene (PS)/9,10-phenanthrenequinone (PQ) on silicon wafer. The illumination LED light (λ_i) is 405 nm and the Bragg peak of the formed OM structure is 615 nm. Error bars are S.D. across 3 samples.

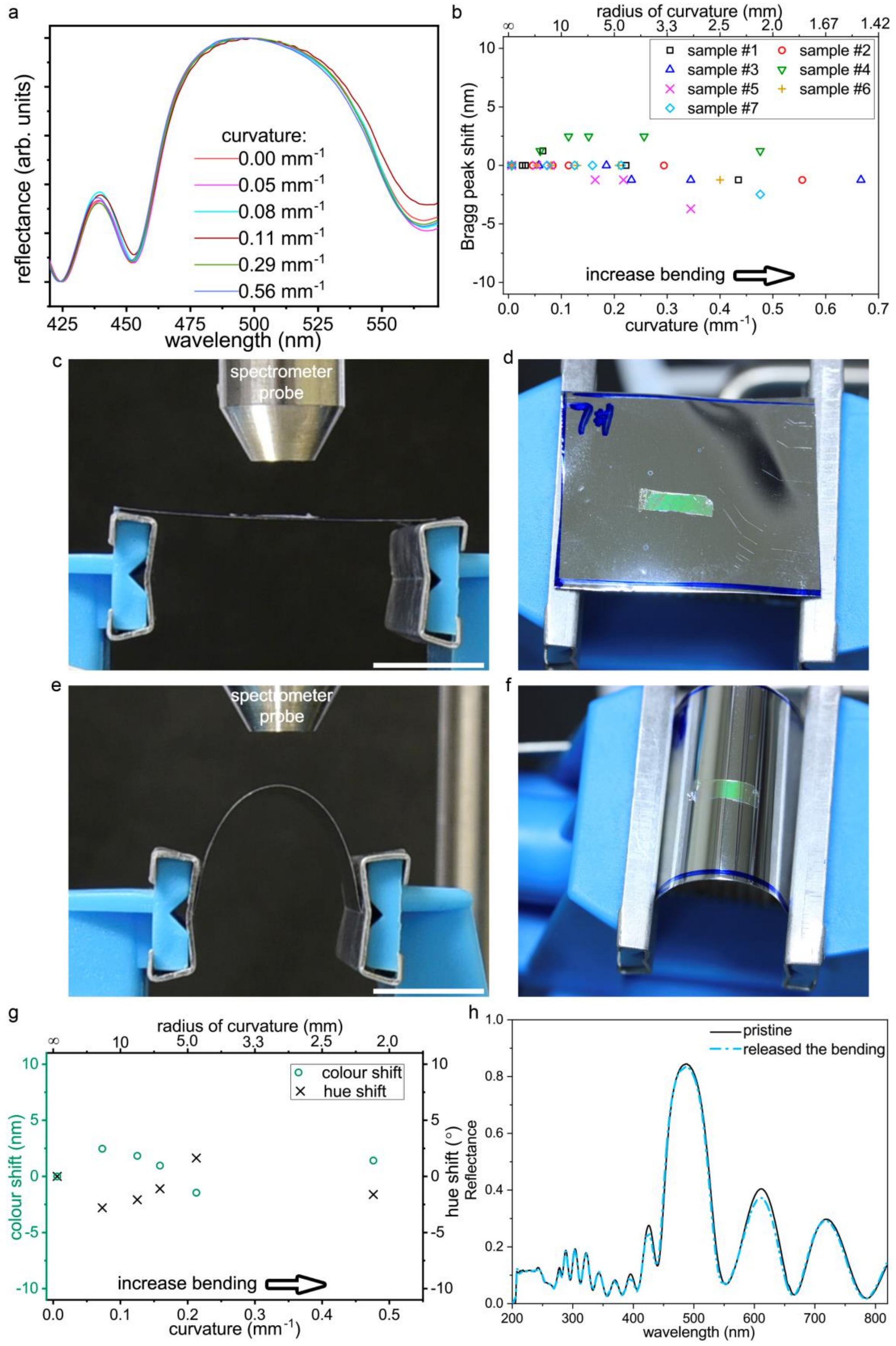

Supplementary Fig. 9 | Impact of bending on the structural colour of OM film. **a**, In situ reflectance spectrum of the OM film during the bending test. The spectrum reference was aluminium (LUXAL UV, 60-μm-thick, Toyo Aluminium K.K.). **b**, Bragg peak shift versus the curvatures of OM film. **c**, **d**, Side-view and top-view photos of an OM film on the aluminium foil before the bending test. **e**, **f**, Side-view and top-view photos of the film during the bending test. **g**, Hue and colour shift versus the curvatures of OM film. **h**, In the end, bending was released and the OM film was placed back onto silicon wafer for spectral

comparison. Scale bar in (**c**) and (e), 10 mm. The OM films were made using Polycarbonate (PC)/4,4′-bis-(diethylamino)-benzophenone (BDABP) on silicon wafer (LED λ_i = 385 nm). The film was peeled off from the substrate carefully in water and transferred onto a piece of aluminium foil. A vice was used for the bending to apply various curvatures to the OM film. As the bending was increased, the in situ reflectance spectrum was recorded. Side-view photos were taken at the same time to obtain the curvature. The curvature was calculated by the software *Fiji* (version 2.1.0/1.53c) using the built-in plugin *Kappa*. Reported values are the average curvature of a 2.5-mm-length curve at the apex region of the bent film where its reflectance spectrum was recorded. In situ measurements show little spectral change when the curvature of OM film is ≤ 0.1 mm^{-1} (radius of curvature: ≥ 10 mm). As the curvature further increases to 0.5 mm^{-1} (radius of curvature decreases to 2 mm), the Bragg peak shift of OM film remains in the small range of 1–4 nm.

As an alternative approach to check the impact of bending, top-view photos were taken for colour comparison under different bending conditions. To keep consistency, an 18% neutral grey card was used as the colour reference. Colour comparison focused on the apex region of the bent OM film that held a fixed angle to the camera lens. The wavelength value of the colour is derived from the hue value according to the visible light spectrum chart, using *ImageJ* software (version 1.52p). Photo comparison indicates that the colour shift of OM film keeps < 3 nm as its curvature increases to 0.5 mm^{-1}. This is consistent with the results from the spectral measurements.

The results show negligible change of structural colour in this range of bending conditions. In addition, after releasing the bending and placing the OM film back onto silicon wafer, the reflectance spectrum remains almost unchanged in comparison to its pristine status. This indicates bending to such an extent does not permanently deform the OM structure.

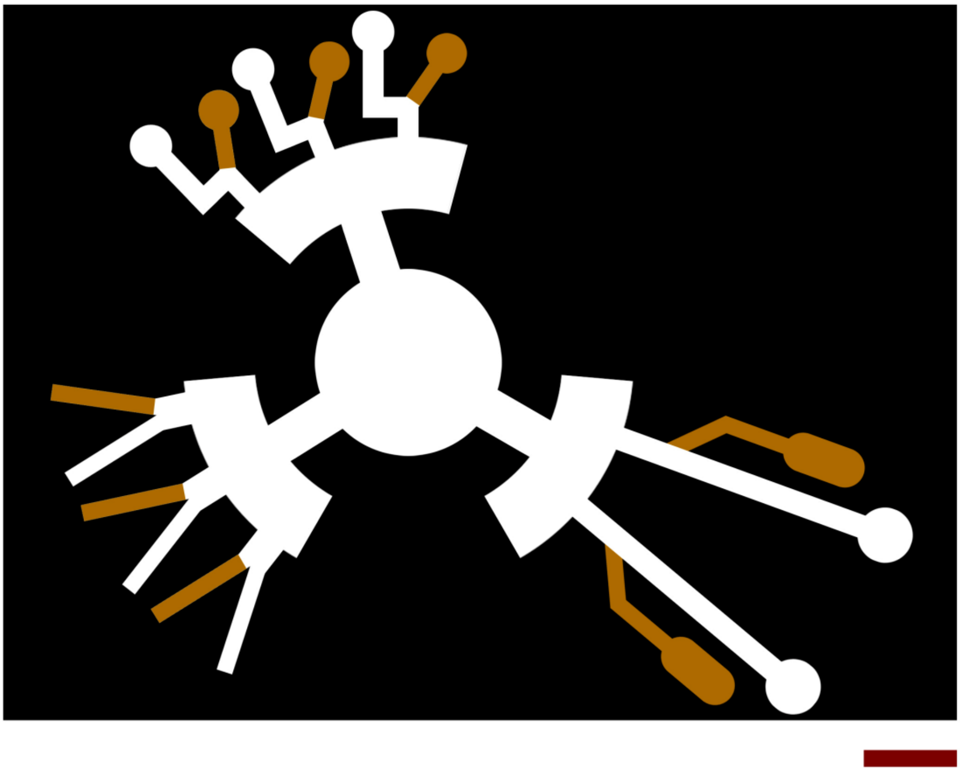

Supplementary Fig. 10 | Energy dosage design for the separation microchannel. The energy dosage is 600 J/cm^2 for the main channel (as indicated by the white colour) while 300 J/cm^2 for the selected side branches (as indicated by the brown colour). In addition, an energy transition region of 32 μm length is applied at the boundary region of the high and low dosage regions. In each transition region, the energy dosage increases gradually by 60 J/cm^2 per 8 μm length from the 300 J/cm^2 side branch to the 600 J/cm^2 main channel. Scale bar, 200 μm. Experiments found that the regions receiving high energy dosage develop faster than the regions receiving low energy dosage during the development step. This is due to the positive relationship between crosslinking energy and the driving force for the development step[2]. Completing the development step based on the high energy regions, the resulting OM structure is found to be fully developed in the high energy regions while not fully developed in the low energy regions. This can generate the final OM microchannel that combines differing internal porosities in a single miniature device.

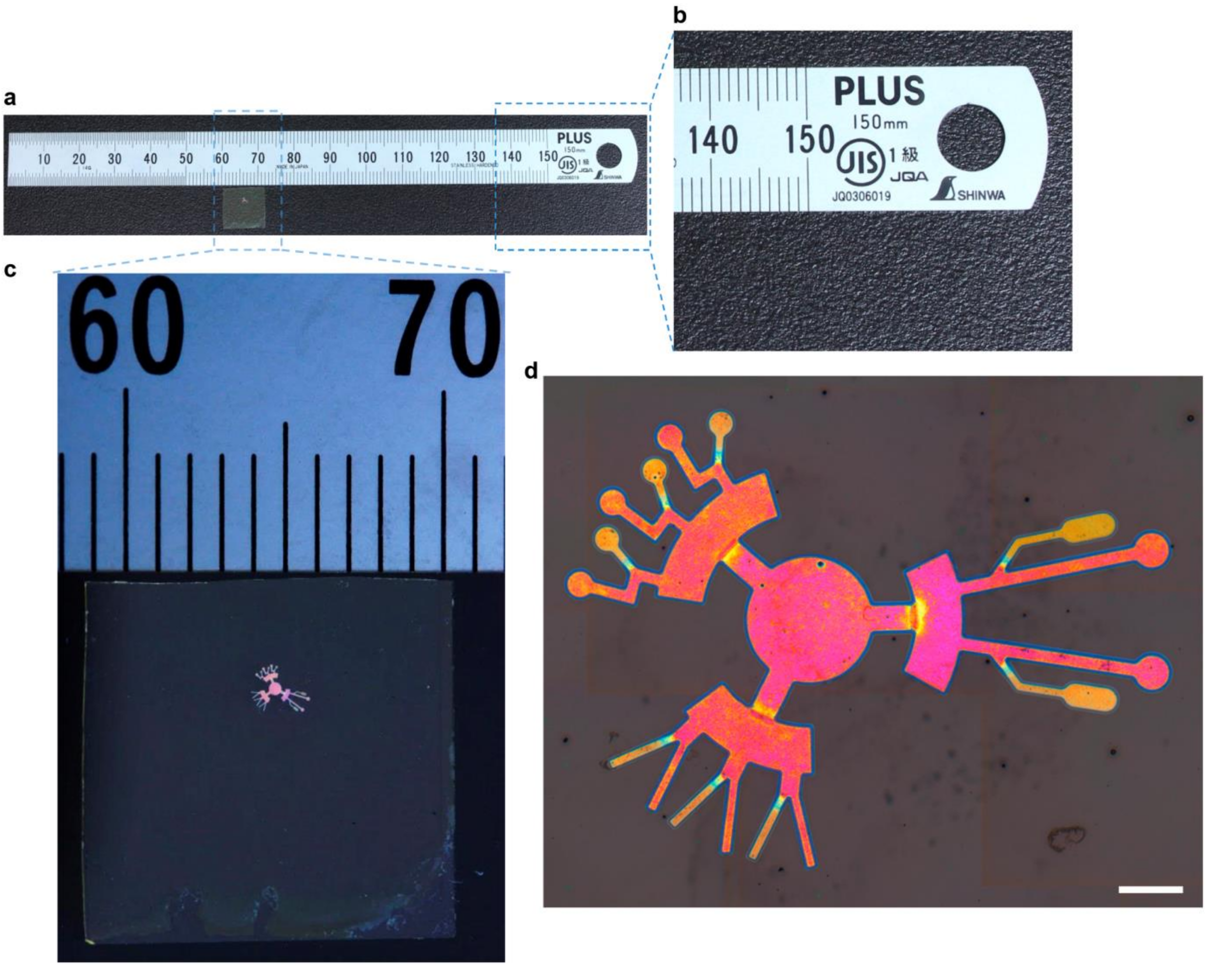

Supplementary Fig. 11 | Photos of OM microchannel on cover glass. a, **b,** Photos of the OM microchannel next to a ruler, taken with a macro lens on DSLR camera. **c**, An enlarged view of the photo in (a), clearly showing the scale of the OM microchannel. **d**, The corresponding microscope photo for the same OM microchannel in (**a**, **c**). Scale bar, 200 µm. This OM microchannel was made on glass substrate using a micro-LED instrument (λ_i = 405 nm). It adopted the energy dosage design in Supplementary Fig. 10 for the illumination step.

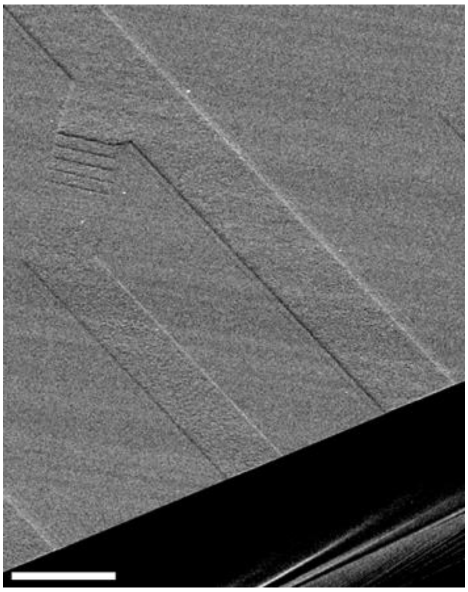

Supplementary Fig. 12| The SEM image for separation channel. It is the original SEM image for Fig. 5f. Scale bar, 50 μm.

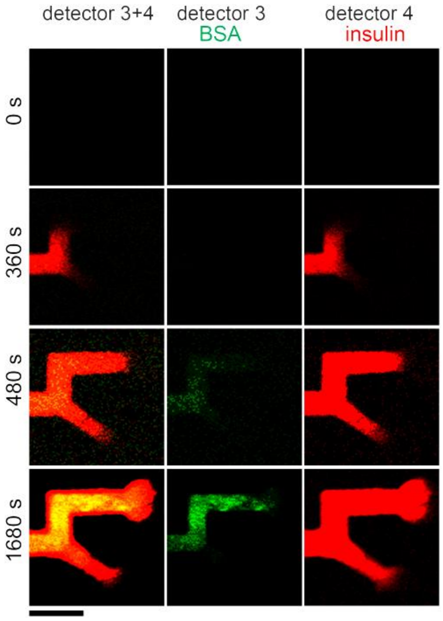

Supplementary Fig. 13| Insulin–BSA separation by the OM microchannel. Imaging was conducted by an inverted confocal laser scanning microscope. The green and red colours represent the BSA protein (66 kDa, pre-stained with the dye SYPRO Orange) and fluorescent insulin (Alexa Fluor 680 labeled, 6 kDa), respectively. The OM microchannel was made using polystyrene (PS)/9,10-phenanthrenequinone (PQ) on cover glass using a micro-LED instrument (λ_i = 405 nm). The microchannel was printed according to the design in Supplementary Fig. 10 with different energy dosages in the main channel and the side branch. The microscope photo of OM channel is shown in Fig. 5e. Scale bar, 100 μm.

Supplementary Table 1 | Experimental details for film casting.

OM channel	Polymer	Photoinitiator	Solvent	Polymer : PI : Solvent in casting solution	Typical thickness of as-cast films (nm)
Macrochannel	PS	BDABP	$CHCl_3$	15:1:284	850 ± 100
		PQ	CH_2Cl_2	7.2:1:135.8	
	PMMA	BDABP	$CHCl_3$	15:1:284	
	PC	BDABP	$CHCl_3$	15:1:284	
Microchannel	PS	BDABP	$CHCl_3$	15:0.67:284	
		PQ	CH_2Cl_2	7.2:1:135.8	

Note: PS denotes polystyrene, PMMA denotes poly(methyl methacrylate), PC denotes polycarbonate, PQ denotes 9,10-phenanthrenequinone, BDABP denotes 4,4’-bis-(diethylamino)-benzophenone.

Supplementary Table 2 | Details for OM channel fabrication.

OM channel code	Scale	Photocrosslinking approach			Polymer	Photoinitiator	Substrate
		Instrument	Photomask	λ_i (nm)			
Fig. 1c	Macro	custom oven	OHP sheet	375	PS	PQ	PET sheet
Fig. 1d	Macro	custom oven	OHP sheet	375	PS	PQ	cover glass
Fig. 1e	Macro	custom oven	OHP sheet	385	PS	PQ	silicon wafer
Fig 1f–h	Micro	micro-LED	N/A	405	PS	PQ	silicon wafer
Fig. 2a,b†	Micro	micro-LED	N/A	405	PS	BDABP	silicon wafer
Fig. 2c	Macro	custom oven	OHP sheet	385	PS	PQ	cover glass
Fig. 2d	Micro	micro-LED	N/A	405	PS	PQ	cover glass
Fig. 3a, b	Micro	micro-LED	N/A	405	PS	BDABP	silicon wafer
Fig. 3c	Marco	custom oven	steel stencil	340-405	PS	PQ, BDABP	silicon wafer
Fig. 3d	Marco	custom oven	steel stencil	340-405	PS	PQ, BDABP	silicon wafer
	Micro	micro-LED	N/A	405	PS	BDABP	silicon wafer
Fig. 3e‡	Marco	custom oven	steel stencil	405	PS	PQ	silicon wafer
Fig. 4a	Micro	micro-LED	N/A	405	PS	BDABP	silicon wafer
Fig. 4b–d	Marco	custom oven	steel stencil	385	PS<br>PMMA	BDBAP	silicon wafer
Fig. 4e	Macro	custom oven§	N/A	405	PC	BDABP	silicon wafer
Fig. 5, 6	Micro	micro-LED	N/A	405	PS	PQ	cover glass

Note: The micro-LED machine does not require a separate photomask.

†The Bragg peak of the tested microchannels is estimated to be 540 ± 10 nm, according to the calibration plot shown in Supplementary Fig. 6.

‡The Bragg peaks of the tested macrochannels are 670 ± 20 nm, measured by spectrometer.

§Light source for custom crosslinking ovens is Thorlabs LED, except Fig. 4c where a Thorlabs Laser is used.

Supplementary Table 3 | Additional details for OM channels that are employed for flow dynamics study.

OM channel code	Channel width	Pattern geometry	Crosslinking instrument	Photoinitiator	Measurement approach
Fig 3a, b	down to 20 μm	bars	micro-LED	BDABP	microscope
Fig 3c	3 mm	straight line	custom oven	PQ	DSLR
Fig 3d, Macro - Series 1	3 mm	straight line	custom oven	PQ	DSLR
Fig 3d, Macro - Series 2	3 mm	straight line	custom oven	PQ	microscope
Fig 3d, Macro - Series 3	5 mm	straight line	custom oven	BDABP	DSLR
Fig 3d, Macro - Series 4	5 mm	straight line	custom oven	BDABP	microscope
Fig 3d, Micro - Series 5	down to 20 μm	bars	micro-LED	BDABP	microscope
Fig 3d, Micro - Series 6	down to 5 μm	vessel, complex	micro-LED	BDABP	microscope
Fig 3e†	3 mm	straight line	custom oven	PQ	DSLR microscope

Note: The pattern geometry of OM microchannels for flow dynamics study are demonstrated in Supplementary Fig. 5b: "vessel" refers to a capillary vessel like pattern, and "complex" refers to a collection of miniaturized microfluidic features including a hexagonal lattice, width variation, spiral, and zig-zag channels. DSLR stands for a digital single-lens reflex camera.

†In Fig. 3e, the flow of pure alkanes was measured by a DSLR camera (EOS Kiss X5) with a macro lens (EFS 60 mm, Canon), while the flow of alkane mixture, pure alcohol, and alcohol mixture was measured by an optical microscope (Axioscope A1 MAT, Carl Zeiss).

Supplementary Table 4 | Viscosity of *n*-decane and *n*-hexadecane binary mixture.

n-decane mole fraction	*n*-hexadecane mole fraction	Viscosity (mPa s)	$Viscosity^{-1}$ ($mPa^{-1}\ s^{-1}$)
0%	100%	3.47	0.29
10%	90%	3.04	0.33
30%	70%	2.33	0.43
50%	50%	1.79	0.56
70%	30%	1.38	0.73
80%	20%	1.20	0.83
100%	0%	0.92	1.08

Note: the viscosity values of pure alkanes are obtained from the literature[3]. The viscosity of alkane mixtures is calculated according to the classic Grunberg-Nissian equation[4].

Supplementary Table 5 | Viscosity of ethanol and *n*-hexanol binary mixture.

ethanol mole fraction	1-hexanol mole fraction	Viscosity (mPa s)	Viscosity^{-1} (mPa^{-1} s^{-1})
0%	100%	4.59	0.22
30%	70%	2.97	0.34
50%	50%	2.22	0.45
70%	30%	1.66	0.60
80%	20%	1.44	0.70
90%	10%	1.24	0.80
100%	0%	1.07	0.93

Note: the viscosity values of pure alcohols are obtained from the literature[5]. The viscosity of alkane mixtures is calculated according to the classic Grunberg-Nissian equation[4].

Supplementary Table 6 | Details for confocal laser scanning microscope experiments.

	Solvent	Fluorescent solutes	The microscope detector used	Other components in the solution
Fig. 2a	ethanol/water (v/v, 9/1)	ATTO 495 (Ex/Em: 495/527 nm), conc.: 0.01 wt%	Detector 2	N/A
Fig. 2b	ethanol/water (v/v, 9/1)	ATTO 610 (Ex/Em: 615/634 nm), conc.: 0.01 wt%	Detector 3	N/A
Fig. 2c	ethanol/water (v/v, 9/1)	ATTO 495 (Ex/Em: 495/527 nm), conc.: 0.01 wt%<br>ATTO 610 (Ex/Em: 615/634 nm), conc.: 0.01 wt%	Detector 2<br>Detector 3	N/A
Fig. 2d	water	GFP (Ex/Em: 475/505 nm), conc.: 0.5 mg/ml	Detector 2	5 wt% acrylamide, 0.1 wt% SDS, 1× PBS
Fig. 6a	water	3-kDa dextran (Ex/Em: 400/420 nm), conc.: 30 μM<br>70-kDa dextran (Ex/Em: 595/615 nm), conc.: 30 μM	Detector 1<br>Detector 3	5 wt% acrylamide, 0.1 wt% SDS, 1× PBS
Fig. 6b	water	3-kDa dextran (Ex/Em: 400/420 nm), conc.:30 μM<br>RFP (Ex/Em: 559/611 nm), conc.: 30 μM	Detector 1<br>Detector 3	5 wt% acrylamide, 0.1 wt% SDS, 1× PBS
Fig. 6c	water	SARS-CoV-2 N (Ex/Em: 470/570 nm)†, conc.:30 μM<br>Insulin (Ex/Em: 679/702), conc.:30 μM	Detector 3<br>Detector 4	5 wt% acrylamide, 0.1 wt% SDS, 1× PBS
Fig. S12	water	BSA (Ex/Em: 470/570 nm)†, conc.: 30 μM<br>Insulin (Ex/Em: 679/702), conc.: 30 μM	Detector 3<br>Detector 4	5 wt% acrylamide, 0.1 wt% SDS, 1× PBS

Note: the confocal laser scanning microscopes (CLSM) are equipped with four lasers (410, 489, 561, 638 nm) and the four associated detectors. Each detector records the emission spectrum of a different wavelength range (detector 1: 425–475 nm, detector 2: 500–550 nm, detector 3: 570–620 nm, detector 4: 663–738 nm). A particular detector with the strongest signal was selected for each fluorescent dye or biomolecule. Biomolecules with distinct fluorescence colour were selected for making mixture solutions. The differentiation of biomolecules in the mixture can be achieved by using multiple detectors simultaneously.

Fresh solutions were used for all the CLSM experiments. The experiments were conducted at room temperature.

GFP denotes a green fluorescent protein. RFP denotes a red fluorescent protein. SARS-CoV-2 N denotes a Severe acute respiratory syndrome coronavirus 2 (SARS-CoV-2) nucleocapsid protein. BSA denotes Bovine Serum Albumin.

† SARS-CoV-2 nucleocapsid protein and BSA were pre-stained with the dye SYPRO Orange (Ex/Em: 470/570 nm) in order to have the fluorescence signal. The pre-staining was conducted by mixing either SARS-CoV-2 nucleocapsid protein or BSA with SYPRO Orange in the aqueous solution (SYPRO Orange concentration was 10× in this mixture). 20 min later, the fluorescent insulin was added for preparing the final test solution.

Supplementary Discussion

Mathematic models for capillary flow dynamics in OM channel

For an incompressible and Newtonian fluid, Hagen-Poiseuille law gives the pressure drop (*ΔP*) of laminar flow through a long pipe of constant cross-section[6,7], as shown in Supplementary Equation (1):

$$\Delta P = \frac{128\mu l Q}{\pi D_h^4} \quad (1)$$

where μ is the dynamic viscosity of the liquid, *l* is the actual flow distance, Q is the volumetric flow rate, D_h is the hydraulic dynamic diameter of the pipe cross-section.

For a cylindrical tube, the cross-section is a perfect circle so that D_h equals to $2r$. Therefore, Supplementary Equation (1) can be rewritten as:

$$\Delta P = \frac{8\mu l Q}{\pi r^4} \quad (2)$$

where *r* is the radius of cylinder cross-section.

In reality, the cross-section of porous nanomaterials is often not a perfect circle. Hence, the effect of cross-section geometry should be included. Moreover, the effective viscosity of liquid (μ_e) should be considered, since the value when liquid is in nanoscale confinement can be significantly higher than the bulk viscosity[8]. The effective liquid viscosity is influenced by liquid-wall interactions at nano scale[9-11]. Hence, Supplementary Equation (2) is modified as follows for an actual porous material:

$$\Delta P = \frac{8\mu_e l Q}{\pi(\alpha r_e)^4} \quad (3)$$

where μ_e is the effective viscosity of the liquid in the porous material, α is a dimensionless geometrical correction factor, and r_e is the equivalent radius. According to the previous studies, $\alpha = 1$ when cross-section is a perfect circle; $\alpha = 1.094$ when cross-section is a square, and $\alpha = 1.186$ when cross-section is an equilateral triangle[12].

The above equation only considers a single channel. An actual porous material can be considered as a collection of many sub-channels with geometrically varying cross-sections. Hence, a further modification of the equation is shown as follows:

$$\Delta P = \frac{8\mu_e l Q}{\pi\,(\alpha r_a)^4} \quad (4)$$

where r_a is the average radius of all sub-channels, and α is the corresponding average value for the geometrical factor.

Capillary flow through such kind of porous material is subject to the combined effects of capillary pressure (P_c), hydrostatic pressure (P_h), and atmospheric pressure (P_a)[13,14], as shown in Supplementary Equation (5).

$$\Delta P = P_c + P_h + P_a \quad (5)$$

When pore sizes are in the micro/nano small scales, capillary pressure (P_c) usually plays

the dominant role compared to hydrostatic pressure (P_h). Meanwhile, P_a equals to 0 when both channel ends are open to the atmosphere. Therefore, Supplementary Equation (5) can be reasonably simplified as follows:

$$\Delta P = P_c \tag{6}$$

According to Young-Laplace law, the capillary pressure (P_c) of the channel can be expressed in Supplementary Equation (7)[15,16]:

$$P_c = \frac{2\gamma cos\theta}{\alpha r_a} \tag{7}$$

where γ is the surface tension of the liquid, θ is the contact angle of the liquid on the surface of the channel.

Substituting Supplementary Equation (4) and (7) into Supplementary Equation (6) obtains Supplementary Equation (8):

$$\frac{8\mu_e lQ}{\pi\,(\alpha r_a)^4} = \frac{2\gamma cos\theta}{\alpha r_a} \tag{8}$$

Volumetric flow rate (Q) equals to $A \times v_\tau$, where A is channel cross-section area and v_τ is the actual flow velocity considering the tortuosity (τ). When channel cross-section is a constant of $\pi \times r_a^2$, Supplementary Equation (8) can be rearranged as follows:

$$lv_\tau = \frac{\alpha^3 r_a \gamma cos\theta}{4\mu_e} \tag{9}$$

The flow path in a real porous media is often not a straight line at small scales; thus, tortuosity (τ, $\tau = l/L$) should be included for the consideration[17]. In our study, L is the observed flow distance recorded by a microscope or DSLR camera, while τ is the tortuosity of the actual flow pathway at nanometer scale. Substituting actual flow distance (l) and velocity (v_τ) with the observed flow distance (L) and velocity (v, $v= v_\tau/\tau$), Supplementary Equation (9) can be rewritten as follows:

$$Lv = L\frac{dL}{dt} = \frac{\alpha^3 r_a \gamma cos\theta}{4\mu_e \tau^2} \tag{10}$$

The observed flow velocity (v) can be written as a differential function of dL/dt. The integration of the Supplementary Equation (10) obtains Supplementary Equation (11), which is the same equation shown in the main text:

$$L^2 = \frac{\alpha^3 r_a \gamma cos\theta}{2\mu_e \tau^2} t \tag{11}$$

Supplementary Equation (11) is a specific form of Lucas-Washburn model for capillary flow through a media with fluidically relevant pore size at nanometer scale. Hence, this equation is suitable for capillary flow in OM channels. It indicates that the observed flow distance square (L^2) is proportional to time (t). The slope of L^2 versus t (dL^2/dt) is the fluidic parameter describing how fast the liquid is spreading in the porous media. In the case of the OM channel, dL^2/dt is proportional to r_a, the internal porosity size of the OM structure. Since the internal pore size is also related to interlayer spacing, the fluidic property of OM channels

is determined by their structural colour (Bragg peak), not the extrinsic channel geometries as printed.

Mathematic models for capillary flow dynamics in conventional hollow channels

In hollow channels fabricated by conventional lithographic techniques, flow dynamics is determined by the extrinsic channel geometries (*i.e.* printed channel width). In an ideal case of the cylindrical tube (τ = 1, α = 1) with negligible liquid–wall interaction ($\mu_e = \mu$), the equation describing flow dynamics in such hollow channels can be expressed as follows.

$$\frac{dL^2}{dt} = \frac{\gamma cos\theta}{2\mu} r \qquad (12)$$

Supplementary Equation (12) is a general form of Lucas-Washburn model of capillary flow dynamics[18]. In Supplementary Equation (12), *r* refers to hollow channel radius, which is associated with extrinsic channel geometries. This equation is applicable for channel radius in or above micrometer scales where liquid – wall interaction is less influential relative to a nanochannel or the channel with nanoscale interior porosity. Therefore, for conventional hollow channels, fluidic property (dL^2/dt) is proportional to extrinsic channel radius (*r*) and thus reducing channel radius by 50% would halve the fluidic parameter dL^2/dt.

Supplementary References

1 Guo, J. J. *et al.* Highly Stretchable, Strain Sensing Hydrogel Optical Fibers. *Adv. Mater.* **28**, 10244-10249 (2016).

2 Ito, M. M. *et al.* Structural colour using organized microfibrillation in glassy polymer films. *Nature* **570**, 363-367 (2019).

3 Griesbaum, K. *et al.* Hydrocarbons. *Ullmann's Encyclopedia of Industrial Chemistry* (2000).

4 Grunberg, L. & Nissan, A. H. Mixture law for viscosity. *Nature* **164**, 799-800 (1949).

5 Garcia, B., Alcalde, R., Aparicio, S. & Leal, J. M. The N-methylpyrrolidone-(C-1-C-10) alkan-1-ols solvent systems. *Phys. Chem. Chem. Phys.* **4**, 1170-1177 (2002).

6 Whitaker, S. Flow in porous media I: A theoretical derivation of Darcy's law. *Transport in porous media* **1**, 3-25 (1986).

7 Loudon, C. & McCulloh, K. Application of the Hagen-Poiseuille equation to fluid feeding through short tubes. *Ann. Entomol. Soc. Am.* **92**, 153-158 (1999).

8 Bocquet, L. & Charlaix, E. Nanofluidics, from bulk to interfaces. *Chem. Soc. Rev.* **39**, 1073-1095 (2010).

9 Feng, D., Li, X. F., Wang, X. Z., Li, J. & Zhang, X. Capillary filling under nanoconfinement: The relationship between effective viscosity and water-wall interactions. *Int. J. Heat Mass Transf.* **118**, 900-910 (2018).

10 Haneveld, J., Tas, N. R., Brunets, N., Jansen, H. V. & Elwenspoek, M. Capillary filling of sub-10 nm nanochannels. *J. Appl. Phys.* **104**, 014309 (2008).

11 Kuo, J. N. & Lin, Y. K. Capillary-Driven Dynamics of Water in Hydrophilic Microscope Coverslip Nanochannels. *Jpn. J. Appl. Phys.* **51**, 105201 (2012).

12 Cai, J. C., Perfect, E., Cheng, C. L. & Hu, X. Y. Generalized modeling of spontaneous imbibition based on Hagen-Poiseuille flow in tortuous capillaries with variably shaped apertures. *Langmuir* **30**, 5142-5151 (2014).

13 Phan, V. N. *et al.* Capillary filling in closed end nanochannels. *Langmuir* **26**, 13251-13255 (2010).

14 Zhao, C., Zhou, J. J. & Doi, M. Capillary filling in closed-end nanotubes. *Chin. Phys. B* **27**, 024701 (2018).

15 Hassanizadeh, S. M. & Gray, W. G. Thermodynamic basis of capillary pressure in porous media. *Water resources research* **29**, 3389-3405 (1993).

16 Liu, H. & Cao, G. Effectiveness of the Young-Laplace equation at nanoscale. *Sci Rep* **6**, 23936 (2016).

17 Gruener, S., Hofmann, T., Wallacher, D., Kityk, A. V. & Huber, P. Capillary rise of water in hydrophilic nanopores. *Phys. Rev. E* **79**, 067301 (2009).

18 Washburn, E. W. The dynamics of capillary flow. *Phys. Rev.* **17**, 273-283 (1921).
